# Prenatal air pollution exposure and growth: The role of placental mtDNA content

**DOI:** 10.1186/2049-3258-73-S1-P34

**Published:** 2015-09-17

**Authors:** Diana Clemente, Maribel Casas, Carmen Iñiguez, Loreto Santa Marina, Tim S Nawrot, Martine Vrijheid

**Affiliations:** 1 UHasselt, Diepenbeek, Belgium; 2 Centre For Research in Environmental Epidemiology (CREAL), Barcelona, Spain; 3 FISABIO, Valencia, Spain; 4 CIBER de Epidemiología y Salud Pública (CIBERESP), Madrid, Spain; 5 Center for Environmental Sciences (CMK), Hasselt University, Diepenbeek, Belgium

## Background and aims

In recent years, evidence has shown that prenatal traffic-related air pollution exposure influences fetal growth. Changes in mitochondrial DNA (mtDNA) content may represent a biologically relevant endpoint on the mechanisms underlying the association between air pollution and fetal growth restrictions. In this study, we aimed to assess the role of placental mtDNA content on the association of prenatal NO_2_ exposure with fetal growth assessed by ultrasound measurements.

## Methods

In this study, we used 333 mother-newborn pairs from the Spanish INMA study (Sabadell: n = 120; Gipuzkoa: n = 152; Valencia: n = 61). We used temporally adjusted land-use regression models to estimate exposure to nitrogen dioxide (NO_2_). We estimated growth curves for femur length (FL), head circumference (HC), abdominal circumference (AC), biparietal diameter (BPD), and estimated fetal weight (EFW) during pregnancy (weeks 12-20, 12-32 and 20-32). DNA was extracted from placental tissue cells. Relative placental mtDNA content was measured using quantitative real-time polymerase chain reaction.

## Results

Each 10 μg/m^3^ increment in prenatal NO_2_ exposure was associated with a relative decrease in placental mtDNA content of 4.3% (95% confidence interval (CI): -7.4, -1.1%). BPD at 12-32 and 20-32 weeks was significantly associated with prenatal NO_2_ exposure (during weeks 0-12, 0-20, 12-20 and 0-32). HC at 12-32 and 20-32 weeks was significantly associated with prenatal NO_2_ exposure during weeks 20-32 and weeks 0-32. The other fetal growth parameters were not significantly associated with prenatal NO_2_ exposure. Each interquartile range increase in placental mtDNA content was significantly associated with an increase of 5.2% (95% CI: 0.4, 7.8%) in BPD at weeks 20-32.

## Conclusions

Our results give an implication that prenatal air pollution exposure can impair fetal head growth. Furthermore, we showed that placental mtDNA content can play a role in this adverse effect.

